# How to Formalize Different Types of Norms in Multi-agent Systems: A Methodology Focused on the T-Norm Model

**DOI:** 10.1007/s42979-024-03052-4

**Published:** 2024-08-01

**Authors:** Soheil Roshankish, Nicoletta Fornara

**Affiliations:** Institute of Digital Technologies for Communication, Università della Svizzera italiana, via G. Buffi 13, 6900 Lugano, Switzerland

**Keywords:** Normative multi-agent systems, Norms models, Methodology, Norms types, Policy models, Monitoring

## Abstract

In a world where many activities are carried out digitally, it is increasingly urgent to be able to formally represent the norms, policies, and contracts that regulate these activities in order to make them understandable and processable by machine. In multi-agent systems, the process to be followed by a person to choose a formal model of norms and transform a norm written in a natural language into a formal one by using the selected model is a demanding task. In this paper, we introduce a methodology to be followed by people to understand the fundamental elements that they should consider for this transformation. We will focus mainly on a methodology for formalizing norms using the T-Norm model, this is because it allows us to express a rich set of different types of norms. Nevertheless, the proposed methodology is general enough to also be used, in some of its steps, to formalize norms using other formal languages. In the definition of the methodology, we will explicitly state which types of norms can be expressed with a given model and which cannot. Since there is not yet a set of different types of norms that is sufficiently expressive and is recognized as valid by the Normative Mutiagent Systems (NorMAS) community, another goal of this paper is to propose and discuss a rich set of norms types that could be used to study the expressive power of different formal models of norms, to compare them, and to translate norms formalized with one language into norms written in another language.

## Introduction

In a world where more and more activities are performed digitally by humans and software agents using the Internet, it is crucial to be able to *formally* represent the rules, norms, contracts and policies that govern the performance of these activities. These are activities of various types that have an impact on the digital life of citizens such as payments, access to online services and those related to creating, storing, using, exchanging and manipulating the enormous amount of digital resources that exist today, such as data spaces, images, videos, books, articles and so on.

The formal representation of these rules is essential to make them readable and even better understandable by the machine and thus being able to provide useful services to those involved in the rules as parties and to those in charge of checking that they are respected. Indeed, it is important to keep in mind that these norms regulating the actions of autonomous agents cannot always be regimented (for example, it is very difficult to regiment obligations [1]), therefore it is possible for such rules to be violated and in certain applications it is crucial to be alerted whenever a violation occurs or a violation will occur as a result of the execution of a certain action.

Analysing the advantages of a formal specification of norms in more detail, we can see that when norms become machine-readable, it is possible to automatically analyze and query them as it is discussed in [2] where the PrivOnto ontology is used for analyzing 115 privacy policies. For example, it will be possible to search the set of resources on which it is possible to perform certain actions based on the customers’ interests. When a policy is formalized with a model that has an operational semantic, it is also possible to provide services for compliance checking of policies [3–6]. This functionality plays an important role, especially in domains in which the customers’ sensitive data is collected because it helps to create a trustworthy environment for customers, this by providing a monitoring platform that they can use to see whether their privacy policies (norms) are violated or not. For instance, a customer can attach to one picture the prohibition to publish it on a public platform for advertisement and would like to monitor if the actions which are performed on the picture are compliant with this prohibition.

Another reason why it can be useful to specify norms with formal languages is that it may become easier for humans to understand their actual meaning, which is not always as immediate as it should be. For example, during the Covid-19 pandemic, it was not always easy to immediately understand what norms are in effect at any given time in a specific location and whether they entail obligations or prohibitions to perform actions. Another example of norms whose meaning and implications are not always immediately clear to the reader are the various privacy policies that regulate the processing of our data when we browse websites and use social networks. Users often accept such policies in order to use online services often without fully understanding what they mean, this is because they are too long or complex.

A considerable number of different models for the formal representation of norms and policies have been proposed in the academic literature in different fields of research such as the study of Normative Multiagent Systems, Digital Law, Privacy and Security, and Access Control for databases. For some of these models a logic-based semantics or an operational semantics has been proposed. In the approach described in this paper we will focus our attention on a subset of these models that are close to being able to be used in real applications on today’s Web, especially those that make use of standard Semantic Web Technologies, a key characteristic for realizing interoperable systems and perform automatic reasoning with guarantees of decidability of the reasoning process.

One of these models, which owes its importance to being a W3C Recommendation, is the Open Digital Rights Language (ODRL 2.2). ODRL is a policy expression language that is specified through an Information Model^1^ for specifying permissions, prohibitions, and obligations about the usage of digital assets and services. The semantics for the concepts and terms of the ODRL Information Model are specified in the core and in the common vocabularies (that are formalized in an OWL ontology).^2^ Two other models for specifying norms and policies based on semantic web technologies are: the OWL-POLAR framework for semantic policy representation and reasoning [3] and the T-Norm framework for automatic monitoring of norms that regulate time constrained actions [6, 7]. As we will discuss in the rest of the paper, these are two complementary models of norms/policies whose operational semantics (the first computed using SPARQL-DL queries and the latter using production rules) can be used for different purposes: the former to do what-if reasoning (i.e. deducing what happens if an action is performed) and anticipating conflicts between policies; the latter to do monitoring and receive notifications if violations or fulfilments of norms occur.

The papers that introduced these models mainly consisted of presenting the components of the model and exemplifying its use by formalising a few examples of norms or policies. What is missing, however, is a *methodology* that explains what steps should be followed if one wants to start from a norm written in a natural language (e.g., English) and be able to choose the model for its formalization and use it to arrive at the formal specification of the norm, which can then be used to reason about it and to evaluate its fulfillment or violation. In particular, in order to be able to decide which of the available models can be used for the formalisation of a given norm (e.g. a norm regulating access to restricted traffic zones in a city), it would be essential to know which types of norms can be formalised with a given model. But since there is no commonly accepted set of types of norms in the literature, papers presenting a given model do not always specify which types of norms can be expressed with that model and which cannot.

*Contributions*. Starting from these two shortcomings (the absence of a methodology and of a rich list of types of norms), in this paper we will give our contribution to the achievement of the following two goals.

The *first goal* of this paper is to propose a rich set of *norms types* that could be used to study the expressive power of different formal models of norms and to compare them. Knowing that a certain model is or is not capable of expressing certain types of norms is fundamental for deciding which model is the best to adopt in a certain application context. For example, if the activation of a norm has to generate a specific obligation for a particular agent and with a precise deadline, it is necessary to choose a model of norms that allows us to express this temporal constraint and check its satisfaction. Secondly, once it is clear that a certain type of norm can be expressed in both model A and model B, it will also be possible to translate norms written in the first model into norms written in the second one. Thus making systems that use different norms models interoperable. This is a fundamental aspect in today’s world where one software agent must be able to interact with multiple open socio-technical systems without having to be reprogrammed and where a system may need to monitor different set of norms that could be specified using different languages.

*Our second objective* is to propose a *methodology* that can be used by people to formalise norms that are written in a natural language with the chosen norm model. By using the term methodology, we mean a systematic method for dealing with a complex problem by specifying the various steps that need to be taken in order to achieve an end goal. This term is well suited to describing a complex task requiring interdisciplinary skills, in fact it requires a profound knowledge of the meaning of the policy one wants to start from, of the model one wants to use and of its semantics, furthermore since there is no one single formalisation possible it requires at each step to decide which of the alternative approaches to follow. The proposed methodology consists in first understand to which types the norm belongs. This step is important because once we know the types of the norm and assuming that we know for a certain model what types of norms it supports, we will be able to determine which models can be used to formalise the norm we started from. The second and more complex step of the methodology consists in coming to a proper formalization of the norm using the chosen model. In this paper, we will focus mainly on formalizing norms using the T-Norm norm model, this is because it allows us to express a rich set of different types of norms. However, it is important to emphasize that the proposed methodology is general enough to also be used, in some of its steps, to formalize norms using other formal languages that have some similarities with the T-Norm model, such as at least OWL-POLAR and ODRL.

This paper is an extended version of paper [8]. In this paper we introduce the “Type of Norms” section (4) to describe a rich list of possible types of norms that can be found in normative systems. Moreover, referring to this new list of types of norms, we have clarified the steps of the methodology and we have added two subsections to represent norms inducing normative power among agents in multi-agent systems (“Formalizing Norms Inducing Normative Power” section) and a section to explain the formalization of exceptions that are used to specify permissions and exemptions (“Representing Permissions and Exemptions” section).

*Organization*. “Related Works” section discusses the problem of formalizing existing norms using formal models and presents relevant and recent papers presenting models for norms and policies specification in which Semantic Web Technologies have been used. In “Running Examples” section two running examples are introduced. In “Types of Norms” section, we will propose and explain a list of different types of norms. In “The T-Norm Model” section, the T-Norm model is briefly presented. Then in “Methodology” section, the proposed methodology for formalizing norms belonging to different types of norms is discussed. Finally in “Conclusions” section we draw some conclusions.

## Related Works

In the multi-agent systems community, over the past two decades, many models of norms and policies^3^ have been proposed to regulate the behavior of autonomous agents [9, 10]. The Semantic Web and Linked Data community is also another active research community in studying the formalization of policy languages for regulating the use of digital assets [11], or for representing legal rules [12].

Norms, formalized with these models, can usually express various types of normative concepts, the most common of which are the notion of obligation, prohibition, and permission. These models (as will be discussed in this article) are generally capable of expressing only some of the different types of norms proposed in “Types of Norms” section. For example, it may be the case that a model can be used to express obligations with a fixed deadline, but is not able to express an obligation whose deadline must be calculated when the norm expressing the obligation is activated.

Since a list of different types of norms that are commonly accepted by the research community does not yet exist, it is impossible for a paper proposing a model to clearly indicate the types of norms that can be expressed by that model. It is also rare to find papers or tutorials explaining a methodology that must be followed to formalize existing norms using a given formal model. That is a methodology that describes the steps to be followed by a person to choose the model of norms they want to use and guides them through the process of formalizing a norm written in a natural language using the selected model.

A paper that presents a framework and a methodology, termed LOGIKEY, was introduced recently in [13]. LOGIKEY stands for **Lo**gic and **K**nowledge **E**ngineering Framework and Methodolog**y** and it was introduced with the objective to support the practical development of computational tools for normative reasoning based on different formal methods. The LOGIKEY methodology consists of the following steps: (i) selecting a logic (like the dyadic deontic logic); (ii) selecting an ethico-legal domain of interest (like the German road traffic act or the GDPR); (iii) deploying the theory selected in the previous step in practical applications to regulate the behavior of an intelligent autonomous system. This methodology is complementary to the one proposed in this paper. Indeed, the first step of the LOGIKEY methodology, which consists of choosing the logic to be used, bears some similarities with the methodology proposed here in which the choice of the model of norms that can be used to formalise a given norm is discussed. But in the LOGIKEY methodology they use classical higher-order logic (HOL) as their formal framework whereas we propose to use models of norms whose semantics is only partially based on description logic. Moreover, our methodology does not focus on choosing a formalism that is then used for a set of norms but explains how to formalise a set of norms of interest on the basis of their type.

Since we cannot compare the proposed methodology with other similar ones in the literature, we consider it useful to introduce in this section some models of norms that have some similarities with the T-Norm model of norms, which will be mainly used in this paper in the exemplification of the methodology. Thanks to these similarities, the proposed methodology can also be used, in some of its parts, for the formalization of norms using such models. In particular, we will focus in this section mainly on those models that use standard Semantic Web Technologies and/or rule-based systems for the formalization of certain components of the norms model or for reasoning about them.

The first norms models that used semantic technologies were the KAoS framework [14], the REI [15] policy language, and the PRovisional TrUst NEgotiation (PROTUNE) framework [16]. Those approaches are summarized and compared in [17] where the requirements for a policy framework are discussed and the various approaches are categorized discussing whether the policies are public or not. For example, for the public policies, it is possible to use KAOS and REI frameworks as we need just one step evaluation to see if the two policies are compatible. On the other hand, if a policy contains sensitive data, they are required to have *stateful and stateless negotiation* protocols for further security concerns.

A well-known policy language based on semantic web technologies, which is a W3C Recommendation since 15 February 2018, is the Open Digital Rights Language (ODRL 2.2).^4^ It is a policy expression language that can be used to represent permitted, prohibited, and obliged actions over a certain asset. ODRL policies may be limited by constraints (e.g., temporal or spatial constraints). ODRL was originally (in 2001) an XML language for expressing digital rights, that is, digital content usage terms and conditions. ODRL 2.2 is a Policy Language formalized in RDF with an abstract information model specified by an ontology. It has no formal semantics, so compliance checking of policies written with this language cannot be performed automatically. An interesting attempt to give a formal semantic to ODRL 2.1 policies is presented in [11]. Some extensions of ODRL have been proposed to overcome some of its limits. In particular, in [5] an extension of the ODRL Information Model has been proposed together with a set of state machines used for describing the evolution in time of the deontic state of obligations, prohibitions, and permissions. Another extension of ODRL is presented in [18] to model both regulatory policies (in the form of nested permissions, prohibitions, obligations, and dispensations), and business policies via discrete permissions. A policy written with that extension of the ODRL language is then translated into an Institutional Action Language (InstAL) [19] policy and thanks to its formal semantics, expressed in Answer Set Programming, it is possible to automatically check compliance and also provide an explanation of the aspects of the policy that brings to the non-compliance. In [20] a specific use case drawn from the social networks field is used to validate the expressiveness of the ODRL 2.0 model.

Other two interesting proposals of a policy/norm model and framework, which are based on semantic web technologies, are OWL-POLAR [3] and T-Norm [6, 7]. Those policies/norms models and their expressivity will be discussed in “The T-Norm Model” section. An interesting aspect that differentiates the two models is the way in which these models define mechanisms to reason about policies to test whether agents’ behavior satisfies them or not. In the OWL-POLAR a query answering mechanism (DL-safe) has been used to check if any action that happened satisfies the policies. In the T-Norm model, a rule-based approach is used that brings the generation of several deontic relations used to represent obligations and prohibitions generated by the activation of norms. In addition, the T-Norm model makes it possible to formalize the temporal constraints that exist between the activation of a norm and the class of actions regulated by the norm.

Other interesting models of norms that, like the T-Norm model, are rule-based are: the one proposed by Garcia-Camino et al. [21] where rules are operationalized using the JESS a rule engine for the Java platform; and the one proposed in [22] where reasoning on norms is realized with DROOLS a business rule management system. Another interesting proposal of a language for the specification of legal text is the OASIS standard LegalRuleML^5^ [23], which defines a rule interchange language for the legal domain that is formalized using RuleML.

In the models that we used in this paper to represent our methodology (such as the T-Norm model or OWL-POLAR), we assume that norms are already selected and we only need to formalize them. However, there exist norm selection approaches that assume the availability of a collection of norms, emphasizing the consideration of moral values in the selection process. In [24] the authors advance the Normative multi-agent systems literature by formally defining the problem and proposing its encoding as a linear program, making it controllable to automated solutions. The research introduces three norm relationships-generalization, exclusivity, and substitutability and considers norm representation power, cost, and associated moral values as explicit preference criteria. The paper emphasizes the importance of including moral values in decision-making, which is a novel contribution in the context of normative systems. In practical terms, the study’s findings have implications for dynamic organizations or evolving societies that already have norms in force. The decision-making process involves considering existing norms alongside a new set of candidate norms. The proposed optimization-based framework provides a systematic approach to determine the optimal norm system, balancing conflicting objectives and constraints. Overall, the paper contributes to enhancing transparency and decision processes in practical tools, paving the way for future research on norm decision scenarios and the interplay between norms and moral values.

Two years later, in [25] the authors propose a qualitative approach to norm selection, focusing on inferring a norm system ranking based on value preferences. This approach avoids the quantitative approaches of previous methods and introduces a novel method for transforming qualitative preferences into norm system preferences. They make two major contributions: a novel ranking system allowing qualitative assessment of norm systems based on promoted values and an encoding method to solve the norm selection problem as a linear program. Some other studies address the challenge of coordinating multi-agent systems through the synthesis of norms. In [26], a novel mechanism named IRON (Intelligent Robust On-line Norm synthesis mechanism) is introduced and designed for the on-line synthesis of norms. IRON aims to produce conflict-free norms for effective coordination without lapsing into over-regulation, ensuring that the synthesized norms are necessary and concise. The norm synthesis problem, which involves determining a set of norms to avoid conflicting states, has been approached through both off-line and on-line strategies. The off-line approaches focus on synthesizing norms at design time, requiring detailed knowledge of the system. In contrast, on-line approaches, such as norm emergence, aim to regulate a system at runtime. However, these approaches face drawbacks like sensitivity to initial conditions and assumptions about agent collaboration. Against this backdrop, IRON is proposed as an on-line synthesis mechanism that not only ensures the effectiveness and necessity of norms but also addresses over-regulation by generalizing norms. Another framework focused on off-line synthesis is introduced in [27]. This paper explains a framework for the off-line synthesis of Evolutionarily Stable Normative Systems (ESNS) within multi-agent systems. The focus is on coordinating agents in multiple interdependent situations that cannot be easily identified in advance. The framework is based on evolutionary game theory and employs simulations and domain information to automatically enumerate potential conflict situations. Norms are then synthesized through an evolutionary process, leading to sets of codependent norms that effectively coordinate agents in various situations. The paper empirically evaluates the framework in a simulated traffic domain, demonstrating its ability to synthesize ESNSs that successfully avoid conflicts in numerous interdependent traffic situations.

## Running Examples

In the remainder of this paper, we will use various examples of norms to exemplify the types introduced and the procedure leading to their formalization with a given formal model. Two concrete examples will be particularly relevant and are as follows.

The first example (which we call Norm1) is inspired by the law regarding access to limited traffic area in Milan city, its formalization in natural language is: “when an agent enters in the limited-traffic area of Milan, between 7:30 and 19:30, they have to pay 5 euro before 24:00 on the day of entry”.^6^

The second example (which we call Norm2) is the rule that must be followed by libraries in Italy regarding the lending of DVDs, it natural language formalization is: “Italian libraries cannot lend DVDs until 2 years are passed from the distribution of the DVD”.^7^

We are going to use these examples in the following sections as well together with some other original examples that have been used in the related works.

## Types of Norms

As mentioned in the introduction, one of the two objectives of this paper is to introduce a list of the various types of norms that can be found in existing regulatory systems governing human relations from laws, to contracts, to policies for the use of services or resources. Our focus will be on *regulative norms*, i.e. norms that regulate the performance of actions by making them obligatory, prohibited or permitted. In this section we will introduce and explain what characterises these different types of norms.

Being able to define a list of types of norms that is accepted by the scientific community is important because it could lead to the possibility to study which types of norms can be formalised with a certain model and which cannot. Secondly, it could lead to the possibility of recognising that a certain existing rule (e.g. an article of a law) belongs to a specific type and it is important in order to then be able to decide which model to use for its formalisation among all those with which that type of norm can be formalised. For example, if we are able to recognize that a norm is activated by an event and when it is activated generates a specific obligation to perform a certain action before a deadline, it is necessary to choose a model capable of expressing all these characteristics in order to express the norm into a machine-readable format.

Based on our research and the study of related works [3, 9, 10, 28–31], we have identified the following four main types for norms:

1. Norms activated by an event or a state of affairs or both;
2. Norms defining general or specific obligations or prohibitions;
3. Norms expressing a temporal constraint for the actions that regulate;
4. Norms that induce normative powers.

It is important to mention that a norm defined in a certain normative system (like for example the European General Data Protection Regulation (GDPR)) may belong to more than one of the types of norms defined here. It means for example that it is possible that a norm that is activated by an event will create specific obligations characterized by a temporal constraint for the regulated action that should happen before a specific deadline.

### Norms Activated by an Event or State of Affairs or Both

The first important distinction between different types of norms can be made on the basis of the type of condition that must be satisfied in order for a norm to become active.

On one hand, there are norms that are activated by the occurrence of an event belonging to a specific class of events (which is described in the norm). When such an event happens, the activation of the norm creates a specific type of deontic relation that obliges certain agents to do something before a specific point of time (deadline) or prohibits them from doing it for a certain interval of time. For example, Norm1 belongs to this type, in fact when drivers enter the limited traffic area of Milan (activation event), they become obliged to do a payment of a certain amount (regulated action) before the specified deadline (midnight on the day of entry). Norm2 also belongs to this type, in fact, from the moment a DVD begins to be distributed on the market (activation event), libraries are prohibited from lending it (regulated action) for two years (interval of time when the prohibition is active). In both examples, there exists a class of actions (where the class of actions is a subclass of the class of events), such as entering a limited traffic area or the distribution of a DVD on the market, such that when an action belonging to that class is performed, it leads to the activation of the respective norm.

On the other hand, there are norms that are activated when a certain state of affairs is satisfied and deactivated when that state of affairs is no longer satisfied. For instance, if we take into account the following norm discussed in [31] that regulates littering in a public park: “agents should not drop litter in the park as long as a rubbish bin is 50 ms away from them”. This norm is activated for an agent when there is a rubbish bin close enough to it and it is deactivated (or we can say it expires) when the agent moves away from a rubbish bin. In this example, the distance between an agent and the rubbish bin is the condition (or the state of affairs) for becoming obliged to use it or for activating the prohibition to drop litter in the park.

There are also norms that are activated upon the occurrence of an event only if certain states of affairs hold. For example, the obligation to pay for access to a city’s restricted traffic zone only if you enter on weekdays.

In “Methodology” section, we will describe how to model a norm, whether activated by the occurrence of an event or the satisfaction of a state of affairs. We will see how this distinction has implications for the choice of the best model to formalize such norms.

### Norms Defining Specific or General Obligation or Prohibition

When a norm is activated (whether by the occurrence of an event/action or by the satisfaction of a state of affairs), it creates an obligation or a prohibition to perform an action that belongs to a specific class of actions. For example, the obligation to pay a certain amount of money or the prohibition to lend a specific DVD.

The obligation or prohibition created by the activation of a norm can apply either to a *specific* agent, who is called the *debtor* of the specific obligation or of the specific prohibition or to a more *general* set of agents, e.g. all the agents playing a given *role* at the time of the activation (we speak about general obligations or prohibitions).

Norm1 belongs to the first category of norms: when a driver enters a limited traffic zone, the owner of the car (a specific agent known at the time of activation) becomes obliged to make the payment before the deadline, and if the payment is not made on time, the owner of the car is the agent who violates the obligation.

Norm2 belongs to the second category of norms. When a DVD is distributed, the norm is activated and it starts to hold the prohibition for all the Italian libraries to lend the specific DVD until the interval of 2 years elapses. The debtor of the prohibited action (lending DVD) is not a specific agent, it is a set of agents, i.e. the agents who play the librarian role in Italian libraries. When this type of norm is activated, there is no specific agent playing the role of the debtor of the obligation or prohibition. The specific agent becomes known in case of violation of the prohibition because he or she is the actor who performs the prohibited action, i.e. the librarian who lend the DVD before the two years passed.

Therefore we can observe that a norm that creates an obligation (like Norm1) or a prohibition that applies to a specific agent can bring the generation of one fulfillment or one violation. Differently, a norm that creates an obligation or a prohibition (like Norm2) that applies to a group of agents can bring the generation of many violations or fulfillments, e.g. in the DVD example, more than one librarian can violate the prohibition.

The recognition of the difference between these two types of norms is important because there are certain models that can be used to formalize a norm if and only if the debtor of the action regulated by the norm is a specific agent that becomes known at the time of the activation of the norm. Such a model cannot be used for the formalization of those norms whose specific debtor is unknown at the time of their activation.

### Norms Expressing a Temporal Constraint for Regulated Actions

An important and widespread type of norms involves specifying whether a particular action must be carried out (obligations) or prohibited (prohibitions) within a certain time interval. In particular, when the norm expresses an obligation, the obliged action must be performed after the activation of the norm and before a specific instant of time that is called the deadline. In this type of norm, when the deadline is elapsed the obligation can no longer be fulfilled and becomes violated. This type of norm is very common in normative systems because the presence of the deadline allows for an expectation of the behavior of the agents subject to these norms.

When the norm expresses a prohibition, the performance of the regulated action is prohibited from its activation until a certain instant of time, after which the prohibition can no longer be violated and becomes fulfilled. For this type of norm, the deadline for obligations or the end of the time interval in which a prohibition is in force may be calculated based on the time at which the norm is activated. Recognizing that a norm specifies a deadline (when it expresses an obligation) or an interval of time (when it expresses a prohibition) is important because it determines the model of norms that should be used for its formalization.

Norm1 and Norm2 belong to this type of norm. Norm1 expresses that when a driver enters a restricted traffic zone, the time instant of this action is very important for calculating the deadline of the payment obligation. Having an obligation to pay without a deadline would not make sense. Norm2 expresses that the release date of a DVD is the starting point of the two-year prohibition on lending DVDs. It is also possible to have another type of prohibition: the prohibition to perform an action without an associated time interval, in this case the prohibition to perform the action will be active forever.

When rules express an obligation to perform an action within a deadline, it is possible to identify two sub-types of such norms based on whether or not the obligation persists after the deadline has passed as it is discussed in [28]. In a certain type of norm, the deadline for the obligation is such that if an agent does not perform the required actions before the deadline, the obligation is violated and the agent can no longer fulfill the obligation. For instance, if a student submits assignments after the deadline, they will not be graded by the teacher. On the other hand, there are norms in which the obligation persists even if the deadline is not met. In the example of the restricted traffic zone, if a driver does not make the payment by the deadline, it does not mean that they are no longer obliged to pay. Instead, they will receive an additional payment obligation on top of the first one, which is the fine for not meeting the deadline. This latter type of regulations are known as *standing obligations*. In some scenarios, it is also possible that the same obligation with a deadline is repeated over a period of time. We call these deadline obligations as *repetitive obligations*[28]. An example of such obligations are the monthly payments to be made by mobile phone subscribers.

In “Methodology” section we will describe how to formalize norms having temporal constraints in detail.

### Norms that Induce Normative Powers

Another type of norm is the one where the norms are able to represent the *normative power* of agents or with a different name used in literature the *institutionalized power* of agents [29].

In [30] the notion of *normative power* is defined as follows: in a normative system, an agent has a normative power if the agent has the ability to create, modify or delete certain norms, and those changes to the norms are recognized by the other agents. This notion of power has been introduced to represent the fact that an agent can modify only a subset of a normative system. This notion of normative power that focuses on modifying only norms runs the risk of not emphasizing that the notion of normative power is related in general to the creation or modification of the normative state of affairs involving agents, i.e. when one changes their rights or obligations not only their norms [29].

Given that, in many norms models, by activating a norm an agent may be able to create obligations or prohibitions for other agents, this means that certain types of norms are able to give a particular power to the agents playing a certain *role*, i.e. the role that an agent has to play for being able to perform the action that activates the norm. For example, in a certain university, the professors supervising a PhD student (they have the specific role of supervisor) have the normative power to request the student to write a paper for a conference, but they do not have the power to request the student to do the shopping for them. This normative power could be represented by a norm that is activated with the request to write a paper. When such a norm is activated then an obligation for the student is created. In [32] these kinds of norms are called *norms in their role definition*. In this paper when we speak about the particular type of norms that induce normative powers, we are considering this extended notion of normative power.

When one wants to understand whether a particular norm is a norm that induces normative powers, it is important to check whether it has the ability to give normative power to certain agents who play specific roles. One approach to figuring out whether norms induce agents’ normative power or not is to look for the notion of “power” or similar notions in the description of the norms. For example in the norm, “The University has the power to oblige students to participate in lectures”, the word "power" already exists in the formulation of the norm. Therefore, by introducing that norm in a normative system the agents are aware that the university acquires such a normative power to create this obligation for its students.

When the notion of *power* is not explicitly present in the formulation of the norm, distinguishing between the norms that give the agents a normative power and those that do not induce a normative power may become difficult. This is because it is possible that the notion of power is hidden in the description of norms. It means that, despite the absence of the notion of *power* in the description of the norm, if in a norm *Agent A* (usually thanks to their roles) may take an action that creates a deontic relation (an obligation or a prohibition) for another *Agent B* in a multi-agent system and Agent B together with all the other agents in the system recognized that they should fulfill such a deontic relation that is created by Agent A, we say that *Agent A* has a normative power over *Agent B*. This happens for example in the following norm: “When professors ask their Ph.D. students to write a paper for a conference, the students are obliged to do it before the deadline of the conference” where there is not any notion related to power in its description. However, by reading the norm we understand that there is an agent (Agent A) with the role of professor that (by performing the action of asking) may create an obligation for another agent (Agent B) having the role of student. Therefore this norm represents the professors’ normative power who can oblige a student to write a paper.

We will discuss in detail how it is possible to model this kind of norm in different circumstances in “Formalizing norms inducing normative power” section.

## The T-Norm Model

The T-Norm model can be used to formalize a precise and rich set of *types of norms* that regulate the interactions between autonomous agents. Namely (as we will further discuss in the paper) the model can be used to formalize those norms with an activation condition expressible as a class of events; the norms that generate general or specific obligations or prohibitions to perform (or not to perform) actions that can be constrained to happen before something else happens; exceptions to those norms and to obligations and prohibitions (i.e. exemptions and permissions respectively); and norms that induce a normative power. Once a set of norms is formalized using the T-Norm model, a specific *framework* can be used to automatically check if the agents’ behavior conforms or does not conform to the given set of norms. This is done by monitoring the evolution of the state of those sets of norms as time passes, events occur and autonomous agents perform actions. The framework for norms monitoring has been proposed by taking into account the *operational semantics* of the T-Norm model. The model, its operational semantics, and the framework were introduced in [6, 7].

The T-Norm model captures the following intuitive meaning of norms: whenever a particular *activation condition* is satisfied (i.e. an event that belongs to a particular class of events occurs) a *deontic relation* (general or specific) is created for regulating (oblige or prohibit) the performance of a class of actions by certain agents. In turn, every time an action belonging to the *class of the regulated actions* is executed before a certain event happens (for example a certain temporal event representing a deadline) and the deontic relation represents an obligation it will be *fulfilled*, while it will be *violated* if it represents a prohibition. On the contrary, when an action belonging to the class of regulated actions can no longer be performed (for example because the deadline has expired) and the deontic relation represents an obligation it will be *violated*, while if the deontic relation represents a prohibition it will be *fulfilled*.

In order to formally describe such a dynamic behavior, the abstract model of a norm cannot only consist of a set of facts (like it is in many models of norms and policies, e.g. ODRL, OWL-POLAR [3], and the model proposed in [22]). In all these models the intrinsically dynamic nature of norms is described in their semantics or is left to their intuitive meaning described in the text. The T-Norm model allows us to specify how the performance of certain actions or the occurrence of certain events will change the state of the interaction among agents. Therefore the basic building blocks of the T-Norm *Abstract Norm* are *rules* of the form ON...THEN...ELSE.^8^ The *Abstract Norm* has not a pre-defined deontic type, as will be discussed in “Methodology” section, it is those who formally specify a norm who will decide whether the norm activation creates obligations or prohibitions. In the T-Norm model, a generic *Abstract Norm* has the following form:
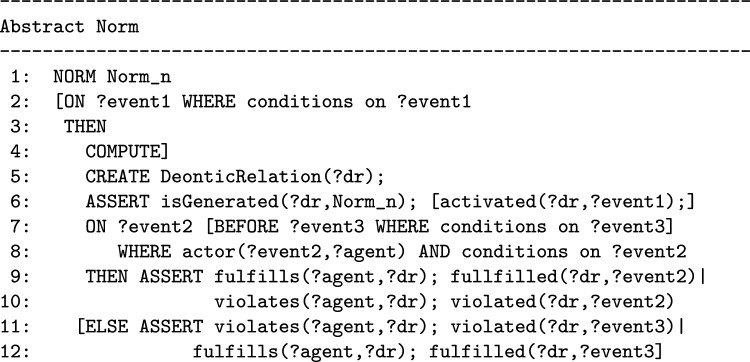


In the proposed model the first (optional) ON...THEN component (lines 2, 3) is used for expressing those norms that have an activation condition. The second ON...THEN component (lines 7, 9) is used for expressing that when a specific action, which belongs to the class of actions regulated by the norm, is performed (before something else occurs) there will be a fulfillment or a violation. In alternative, the ELSE part of the second rule (line 11) will be followed when an action that belongs to the class of the regulated actions can no longer be performed.

**Fig. 1:**
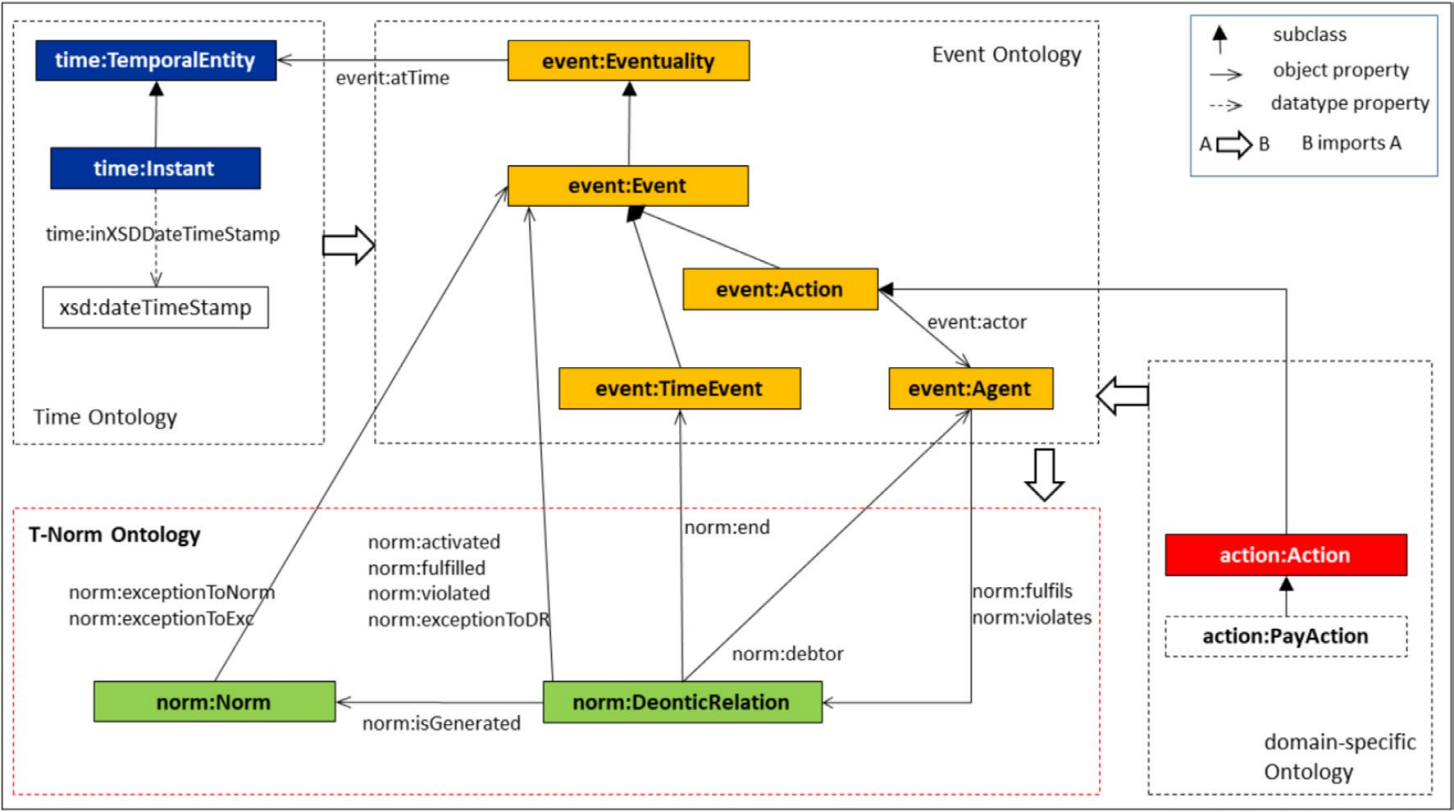
The T-NORM Ontology and its connections with other ontologies

The *formulas* used in the *Abstract Norm* are conjunctions (in the WHERE part) or sequences (in the CREATE and ASSERT part) of *atomic assertions* written using the classes (unary predicates starting with a capital letter) and the properties (binary predicates starting with a lowercase letter) defined in the *T-Norm Ontology* depicted in Fig. 1.^9^ Variables (starting with ‘?’) refers to individuals. Variables used in the WHERE parts of the norm for expressing conditions on events can be used freely and have to be bound to individuals in the *State Knowledge Base* (where the interaction among agents is represented) for the conditions to be met. In the WHERE parts, it is also possible to compare the value of a variable with a constant value using any of the symbols {$<,>,=,\ne ,\le ,\ge$}. A constant is a numerical value or an individual in the ontology. Variables that appear in the ASSERT part of a norm must have been introduced previously in one of its ON or CREATE parts. In the COMPUTE part some values can be calculated (for example the deadlines) using the value of previously introduced variables.^10^

By using the T-Norm model it is possible to formalize exceptions, that can be used for modeling both *permission*, as an exception to prohibitions, and *exemption* as an exception to obligations. By using the model it is possible to specify different types of exceptions. The first is an exception to the activation of norms. When certain peculiar conditions are satisfied on the event activating the norm, the resulting deontic relation has not to be generated. For example “firefighters do not have to pay the train ticket”. The second type of exceptions are those to deontic relations, i.e. when specific conditions on the regulated event are fulfilled the generation of violation/fulfilment is blocked. For example, in a library, the lending of a certain book is prohibited to everyone except teachers. There is also a third type of exceptions, these are those always related to deontic relations (like the second type) but which are triggered by an event that is not the one regulated by the norm. This third type of exception can be formalized only for those norms that generate *specific* deontic relations.

An exception is expressed in one of the following ways on the basis of its type:
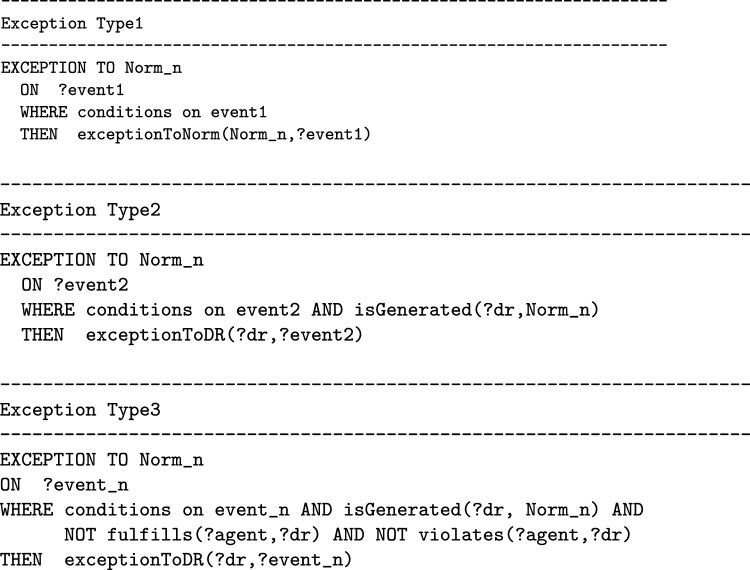


In order for exceptions to inhibit norms, they must be evaluated before the norms. In the T-Norms model where norms and exceptions are translated into production rules to be computed, the rules of exceptions have higher priority than those of norms. In “Representing Permissions and Exemptions” section we will explain how to formalize these types of exceptions in detail by using some examples.

## Methodology

In this section, we describe the various steps of the procedure to be followed to transform a norm written in a natural language (for example in English) into a norm written using a formal machine-readable language like the T-Norm model. As will be discussed, some steps of the described procedure can also be used to formalize norms using the OWL-POLAR model or the ODRL policy expression language. Starting from a norm expressed in natural language, following each step of the methodology, the *Abstract Norm*, introduced in “The T-Norm Model” section, is made at every step more concrete to the point of being the formalization of the norm from which the process started.

### Using Ontologies for Modeling Norms

As discussed in the Introduction, we decided to propose a methodology that can be used to formalize norms by using models based on Semantic Web Technologies. In particular, in those models the activation conditions of the norms, the class of actions that is regulated by the norm, the expiration conditions, and the temporal constraints to the performance of the actions regulated by the norm are specified using the classes (aka concepts) and the properties defined in ontologies encoded using one of the semantic web languages, namely the W3C Web Ontology Language (OWL 2) or RDF-Schema. An important advantage of using those well-known standard languages is that they can easily be used by those who need to formalise norms.

In the **first step** of the procedure, it is required to: *identify the events, the actions, the activation and expiration conditions, and the temporal constraints described in the norm and decide which OWL or RDF-Schema ontologies can be used to represent them*. Such ontologies can be defined from scratch or better, in a re-usability perspective, through the re-use of existing ontologies that can then be adapted to express the concepts and properties present in the norm under consideration.

When the ODRL policy language is used for the formalization of one policy, the terms in the policy can be expressed using the *ODRL Common Vocabulary*^11^ that provides a list of actions and properties for representing statements about the usage of content and services (for example it defines actions like Display, Distribute, Derive and properties like payAmount and spatialCoordinates).

When the norm is formalized using the T-Norm model, three *classes of events or actions* should be specified using ontologies:

- The class of *events* that represent the *activation condition* of the norm. The variable ?event1 in line 2 of the *Abstract Norm* refers to an individual that belongs to the activation condition class and such a class is described in the WHERE part using the classes and properties defined in the adopted ontologies;
- The class of *actions regulated by the norm*. The variable ?event2 in lines 7,8 of the *Abstract Norm* refers to an individual that belongs to the class of actions regulated by the norm. Such a class is described in the WHERE part (line 8) using the classes and properties defined in the adopted ontologies;
- The class of *events* defined for *constraining the performance of the actions regulated by the norm*. One action, belonging to the class of the regulated ones, should or should not occur before an event belonging to this constraining class. The variable ?event3 in line 7 of the *Abstract Norm* refers to an individual that belongs to the constraining class. As in the previous two points, in the WHERE part (line 7) the constraining class is specified using the classes and properties defined in the adopted ontologies.

The ontologies selected for the formalization (each one referred to as a *domain-specific Ontology* in Fig. 1) should be imported into the *Event Ontology* which is formalized using the OWL 2 language. Therefore even if in principle it is possible to use several different ontologies for specifying the class of actions and their properties inside one T-Norm norm, for compatibility reasons, we suggest using ontologies that are compatible with OWL ontologies.

When a norm is formalized with OWL-POLAR policy model, the class of actions regulated by the policy is specified in a similar manner as with the T-Norm model. Due to substantial differences between the two models, in OWL-POLAR it is not required to describe a class of events as activation condition but it is possible to specify a condition on the state of the world (such as “when there is a fire risk” or “if a room contains patients”). Furthermore, it is not required to specify a temporal constraining class of events but it is necessary to specify an expiration condition (such as “there is no longer a fire risk” or “the room is empty”).

We will now exemplify the formalization of the two classes of events necessary for the formalization of Norm1. Norm1 is activated every time a vehicle enters the restricted traffic zone of the city of Milan. We assume that the RestrictedTrafficAreaAccess is an OWL class of actions, vehicle and owner are two properties; the first has a domain RestrictedTrafficAreaAccess class, the second has a domain of class Vehicle. Those classes and properties are defined in an OWL 2 domain-specific Ontology. The class of events that activates Norm1 can be specified in the following way^12^:
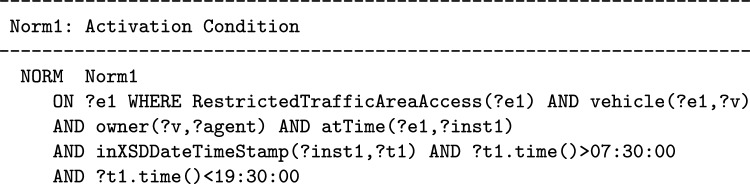


In the previous expression the variable ?agent is introduced because it will be used in the second part of the norm to recognize who fulfills or violates the norm. The properties used to express conditions on when the event occurs are defined in the Time Ontology in OWL^13^ which is imported in the *Event Ontology* as it is represented in Fig. 1.

The class of actions regulated by Norm1 is the payment of 5 euro before 24:00 on the day of entry. For formalizing it we can for example use the PayAction class defined in the *Schema.org*^14^ vocabulary, which has an OWL version. Schema.org represents an interesting attempt to realize a lightweight ontology that can be reused in different applications and in particular it is used for the specification of structured data on the Internet and on web pages and is used by main search engines.

As mentioned earlier, the class of events described with the variable ?event3 has the role of constraining the time interval in which the action belonging to the class of actions regulated by the norm shall or shall not be performed. In Norm1, the time interval when the payment action should be performed is constrained by a deadline (below referred with the variable ?paymentDeadline), i.e. the payment action must occur before 24:00 on the day of entry into the limited traffic area. The formalization of norms where ?event3 is a time event are discussed in “Representing the Temporal Constraints of Norms” section. However, in the formalization of other norms the time constraint could be expressed with any class of actions (e.g. the payment must be made before leaving the restricted area) and in this case we will need to use another ontology to represent that class. The class of actions regulated by Norm1 can be specified in the following way (where ?e1 and ?agent are the variables introduced in the specification of the activation condition of Norm1 in the previous ON clause):
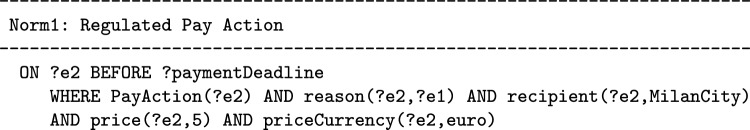


### Formalizing the Activation Condition

The goal of this section is to explain the **second step** of the methodology. It consists in recognizing whether a norm has an *activation condition* or not and if it does, how to formalize it by taking into account its type, i.e. whether the norm is activated by the occurrence of an event that belongs to a *class of events or actions* or by the occurrence of a certain *state of affairs*.

The question to ask in order to recognise whether a rule has a trigger condition is: under what conditions is there an obligation or prohibition for someone to perform a certain action? If these conditions correspond to the occurrence of an event or action then the norm can be modelled with the T-Norm model and it is important to underline that in the T-Norm model, the activation condition of a norm cannot simply be a description of a state of affairs. If these conditions relate to the fulfilment of a certain state of affairs then the norm can be modelled with the OWL-POLAR or ODRL model.

A very common objection to the differentiation between events and states of affairs such as the one proposed here is that an event leads to a new state of affairs and that therefore the new state of affairs can be used as a condition instead of the event. For example the effect of entering in the limited-traffic area of Milan is the state of affairs of being inside the area. But this event-state substitution is not always possible because for certain norms the activating event has crucial characteristics (such as an instant of time when it happens) that cannot be replaced by a state of affairs.

The reason why, in norm models, these two different types of activation conditions are treated differently is mainly due to their ontological difference: an event happens at a specific point in time and when it has happened it can no longer be retracted. Differently, there are states of affairs that can be satisfied for a certain interval of time and become unsatisfied in another interval (as for example the condition “it is a weekday”). This is a crucial difference, indeed in the T-Norm model, any satisfaction of the activation condition leads to the permanent creation of deontic relations. This permanent creation is required when the newly created deontic relation regulates a class of actions that should or should not be performed in an interval of time and when the deontic relation itself can generate many violations and fulfillments, as it is discussed below when Norm2 will be formalized. On the other hand, in the OWL-POLAR and ODRL models, the satisfaction of the activation condition simply leads to an activated policy for a specific agent, and when the activation condition is no longer satisfied or the expiration condition becomes satisfied, the policy becomes inactive.

By using the T-Norm model, in order to recognize the activation condition in the text of a norm, we have to look for the events or actions that when happen induce the model to create certain obligations or prohibitions. When an event that belongs to the class of events described in the activation conditions actually occurs a new *deontic relation* expressing the obligation or prohibition to do a certain class of actions is created and, if applicable, the values of the time constraints relating to the execution of that action are calculated. The temporal relation between an event or action that satisfy the activation condition and the action that should or should not be performed is crucial: the activation condition must be satisfied before the obligation or the prohibition to perform a certain class of actions starts to hold. The instant of time at which the activation condition of a norm is satisfied by an event or action is very important because it can be used to calculate the deadline of obligations generated by the norm or the instant of time at which a prohibition ceases to subsist. For example in Norm1, the activation condition is represented by the class of actions regarding entering the Milan limited traffic zone and it is used for computing the deadline for the payment, its formalization is available in the previous section. In Norm2, the activation condition is given by the class of actions with which a DVD distribution is initiated and can be formalized, using the classes and properties of OWL ontologies, in the following way:
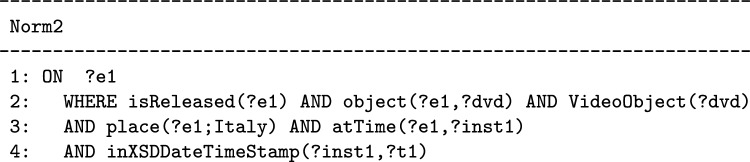


When the activation condition of a policy describes a state of affairs, the OWL-POLAR model can be used for their formalization. Examples of this type of policies are: “a person is obliged to leave a location when there is a fire risk” or “when a person has a child which is under 18 they have to pay their tuition” [3], where the activation condition is the part after the word “when”. In the OWL-POLAR model, a policy is activated for a specific agent when the world state is such that the activation condition holds for that agent and the expiration condition does not hold. Therefore, at the time of activation, it is necessary to know the specific agent for whom the policy is being activated. As we will further discuss in “Formalizing Specific or General Obligation or Prohibition” section, this is not the case when the activation of a policy leads to the creation of *general deontic relations*. Another interesting aspect of the OWL-POLAR policy model, is that in this model there is not an explicit representation of the requirement that the activation condition must be satisfied before a policy could become fulfilled or violated. This requirement is only expressed in the description of the process for reasoning about policies activation.

In the ODRL 2.2 model, the Rule class is the parent of the Permission, Prohibition, and Duty classes and it represents their common characteristics. It is possible to express constraints associated with the Rules contained in one policy and refinements associated with three parts of the rule: the actions regulated by one Rule, its Asset Collection, and its Party Collection. In the ODRL Information Model 2.2 there is a textual specification of the meaning of a prohibition and a Duty, and one can see the satisfaction of constraints can be considered as an activation condition of the rule. Therefore, when the activation condition is a state of affairs, the policy designer can choose whether it is better to put the conditions in the constraint or in the refinement of an ODRL policy. But, as in the case of OWL-POLAR, the time constraints between the satisfaction of the activation condition and the execution of the policy-regulated action are not explicitly expressible with the ODRL model and it is impossible to specify in the policy formalization the how to calculate at run-time the value of deadlines. It is also impossible to formalize those policies that when they are activated generate *general* deontic relations that will be discussed in “Formalizing Specific or General Obligation or Prohibition” section.

By analysing the numerous examples of policies formalised with the ODRL 2.2 model, we made an interesting observation: when the activation condition in the text of a rule describes a state of affairs, one should ask whether this condition should actually be formalised as an activation condition or whether it is better to formalise it as a set of conditions delimiting the class of actions regulated by the policy. For example, consider the following conditional norm “it is prohibited to litter as long as there is a rubbish bin within x meters from an agent” (which is discussed in [31]). The condition of being within x meters of a rubbish bin may be modeled as an activation condition but it can also be considered as a condition that constrains the class of actions regulated by the policy, i.e. littering when the actor of the action is within x meters from the rubbish bin. When this last approach is adopted, the policy can also be modeled with the T-Norm model because there is not anymore a state of affairs as an activation condition. The choice between using the first or second formalization depends on the type of reasoning that the norm designer^15^ wants to be able to perform on that policy. In the first case (when the state of affairs is formalized as an activation condition) it is possible to compute if the policy is active in a given situation and therefore plan the action for fulfilling or violating it. When computing the satisfaction of all the activation conditions of the policies may be too costly or the goal of reasoning about policies is monitoring their fulfillment or violation, the second formalization (without activation condition) is the more efficient because it does not require computing the activation of the policies.

### Representing Obligations and Prohibitions

In this section, we explain the **third step** of the methodology, by clarifying: (i) how to recognize whether a norm generates an obligation or a prohibition; (ii) how to express obligations and prohibitions using one formal model.

By reading the text of a norm it is quite intuitive to recognise its deontic component, usually the verbs “have to” or “must” or “is obligatory” are used to express obligations. Conversely, the negation of these verbs or the verb “cannot” are used to express prohibitions.

Sometimes, however, it may be convenient to formalise a norm containing an obligation as a prohibition and vice versa. In fact, as we know from deontic logic literature [33] the expression “it is impermissible (IM) that *p*” is defined as equivalent to “it is obligatory (OB) that not p” (*IMp* = *def* $OB \lnot p$). In particular, when the activation of a norm brings to the creation of *general deontic relations* (see “Formalizing Specific or General Obligation or Prohibition” section for more details), it is very important to evaluate which of the two formalizations would be most cost-effective. That is because, as discussed below, every general deontic relation created by the activation of a norm, may in turn bring to the costly generation of many fulfillments and violations. For example, the norm “when the school bell rings, students should go back to the classrooms in five minutes” can be formalized either as a norm that generates obligations or as a norm that generates prohibitions. Suppose that the person in charge of formalizing the norm is only interested in computing the violations of the norm. In the first scenario, if we formalize the norm as a generator of obligations when the activation condition is satisfied because the school bell rings, the norm generates a general deontic relation that will generate fulfillments for all those students who respect the school rule and go back to their classrooms, and violations for those students who did not fulfill the rule before the deadline. In the second scenario, it is possible to formalize such a norm as a generator of prohibitions by reframing it as follows “when five minutes have elapsed since the bell rang, students cannot remain in the courtyard”. The formalization of this norm is much easier and cost-effective as we only need to check the violations that are generated for those students who stay in the courtyard.

Once it has been decided whether to formalise a particular norm as an obligation or as a prohibition, if it has been chosen to use the OWL-POLAR or the ODRL model, it is enough to explicitly indicate if the policy is (or contains) an obligation or a prohibition. Differently from those two models, in the T-Norm model there is not a component or a predefined class that may be used to specify whether the norm expresses an obligation or a prohibition. The advantage of this approach is that both obligation and prohibitions can be expressed starting from the same abstract norm and there is no need to formalize the semantics or the state machine for obligations, another one for prohibitions, and others for other deontic concepts like permission, right, privilege, liability and so on, as it is proposed in [5].

In the T-Norm model the intuitive meaning of having an obligation or prohibition is that when something happens and certain conditions hold, an agent is obliged or prohibited to do something in a given interval of time. We can use a few basic constructs and combine them in different ways to express the obligation to perform an action before a given deadline or the prohibition to perform an action within an interval of time. The main difference in formalising a prohibition or an obligation lies in the choice of the fulfills or violates assertions to be included in the last THEN ... ELSE part of the abstract norm presented in “The T-Norm Model” section.

If the norm designer wants to formalize the obligation to perform an action, performing the regulated action must bring to the specification of the fulfillment of the deontic relation by a specific agent in the THEN part of the norm. The ELSE part has to be used to specify that in case an action belonging to the class of actions regulated by the policy cannot be performed before a certain event happens, the deontic relation (representing the obligation) becomes violated. On the contrary, if the norm designer wants to formalize the prohibition to perform an action in a specific interval of time, performing the action will bring to the violation of the deontic relation in the THEN part. Once the prohibited action can no longer be performed before a certain event happens (for example, the time interval has expired) the deontic relation (representing the prohibition) becomes fulfilled.

### Representing the Temporal Constraints of Norms

The **fourth step** of the methodology consists in recognising whether the norm regulates an action that must, or must not, be performed before:

1. an event/action occurs (i.e. “pay before leaving a car park”), or
2. a given instant of time has elapsed (i.e. “pay before the end of year 2023”), or
3. an instant of time, whose value is calculated at the time of activation of the norms, has elapsed (i.e. as in Norm1 “pay before midnight on the day of entry”).

This can be done by searching the text of the norm for the words “before” or “until” or the indication of precise time intervals where the regulated action should or should not be performed.

In order to formally express such a constraint the T-Norm model has to be used. In fact, the ability to represent temporally constrained actions in norms is one of the distinguishing features of the T-Norm model that differentiates it substantially from the OWL-POLAR and ODRL models. Indeed in these two models, it is impossible to express deadlines for obligations, especially those whose value is computed at run-time.

A completely different time constraint that can be expressed in OWL-POLAR is the *expiration condition* of a policy that, when it is satisfied, makes the obligation or prohibition inactive (for example, the obligation to leave a place ends when the risk of fire ceases). Similarly, when ODRL is used, it is possible to formalize a constraint for the performance of an obliged or prohibited action by using the dateTime leftOperand, but its semantics is not well documented and examples of its use express only permission and no obligation or prohibition.

Once it is realised that the T-Norm model must be used to formalise the norm under consideration, it is necessary to proceed by specifying the part of the *Abstract Norm* (see “The T-Norm Model” section) that follows the BEFORE clause. Here it is required to specify the description of the class of events that constrains the performance of the actions regulated by the norm by specifying to which class the variable ?event3 belongs and what the values of some of its properties must be. Based on the type of constraint specified in the norm, it must be specified that the variable ?event3) belongs to the TimeEvent class or the more generic Event class (see Fig. 1).

The TimeEvent class is used for specifying that ?event3 is a time event that happens at a specific instant of time, which represents a deadline for an obligation or the instant of time when a prohibition ends. For example, in Norm1, an agent is obliged to perform the *paying* action before midnight (the deadline) of the day on which the norm was activated. In Norm2, the time interval in which Italian libraries cannot lend DVDs begins with the release of the DVD (activation of the norm) and ends after 2 years. Following all the steps of the methodology explained so far, Norm2 is a prohibition activated by an event that can be represented with the T-Norm model as follows:^16^
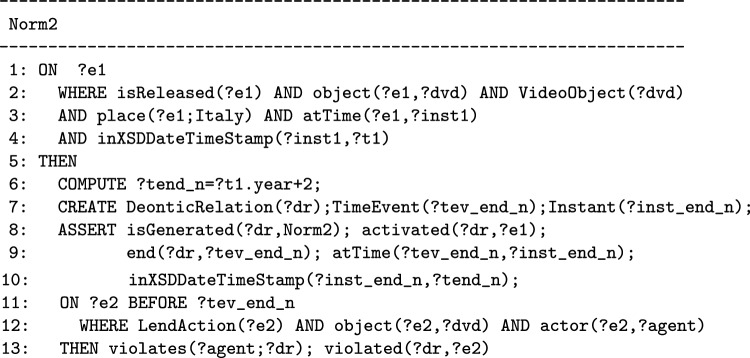


The COMPUTE, CREATE and ASSERT components are important parts of the norm above, which are used to specify the characteristics of the time event used to constrain the class of actions governed by the norm. Their formulation in the above norm represents a prototype of what these components look like in all kinds of norms of this type. In particular, the time event, represented with the variable ?tev_end_n (instead of ?event3), is associated to a specific instant of time by using the atTime property whose value is computed when the norm is activated.

The Event class (or one of its sub-classes) is used for specifying that the temporal constraint of the norm belongs to a generic class of events. This means that the regulated action is temporally constrained by another generic class of events. For example, in the norm “you have to pay the parking ticket before leaving”, there exists no deadline for the payment action, but the payment action must be performed before leaving the parking area with one’s car. Therefore, in the ‘conditions on ?event3’ part of the *Abstract Norm* the event of leaving the parking should be specified.

In literature there exist other models, such as [34], in which time-constrained norms are represented using temporal logics, such as the Linear Temporal Logic (LTL). However, using these approaches present some difficulties when it comes to automatically reasoning on the evolution of the normative state from activated to fulfilled or violated.

### Formalizing Specific or General Obligation or Prohibition

The **fifth step** of the methodology consists in recognizing whether the norm under consideration is a norm that defines *specific* or *general* obligation or prohibition, that were introduced in “Norms Defining Specific or General Obligation or Prohibition” section. Namely, it is important to distinguish between two types of norms: those that can be activated for a *specific* set of agents, this means that at the time of the activation the specific debtor of the activated obligation or prohibition is known; and those that can be activated for a *general* set of agents, whose specific name is unknown at the time of the activation.

The fact that the norm to be formalized belongs to one or the other type has an impact on the choice of the normative model to be adopted. In particular, if the norm belongs to the first type (such as Norm1), both the T-Norm and the OWL-POLAR model can be used; if the norm belongs to the second type (such as Norm2) only the T-Norm model can be used for its formalization. Regarding the ODRL model, it can only be used when the debtor (that is ODRL is called the assignee) of the obligation or prohibition is already known before the policy is activated.

We will now better explain how to proceed in formalising the norm. If the norm defines a *specific* obligation or prohibition and the OWL-POLAR model is chosen, it is important to to include the ?x variable, used in OWL-POLAR to represent the policy addressee, in the policy activation condition as well. This is required because the policy can only be activated for all those *specific* agents for which the activation condition is satisfied. Thus, when the condition is satisfied by the state of the world, there will be one or more substitutions of the ?x variable present in the activation condition with specific agent names.

Differently, if the T-Norm model is adopted, we have that the norm can create many *specific deontic relations* with a known debtor, one, each time its activation condition becomes satisfied by the execution of an action or the occurrence of an event. For example in Norm1, for each vehicle entering into the limited traffic area an obligation to pay for the owner of the vehicle is generated. This is expressed in the formalization of Norm1 by using, in the ASSERT part, the debtor property to connect the generated deontic relation with the owner of the vehicle that entered in the limited traffic area (i.e. by using the following formula: debtor(?dr,?agent)). The debtor property has to be specified in the ASSERT part of the formalization of all those norms that generate specific deontic relations.

If the norm belongs to the second type, only the T-Norm model can be used for its formalization. For example, when Norm2 is activated, i.e. when the DVD is distributed, the activation condition is not satisfied for specific agents. In this case the agents who will be able to violate the prohibition to lend the DVD are the actors of the lending action, i.e. the agents working for Italian libraries. This type of norm cannot be formalized using the OWL-POLAR model because the satisfaction of the activation conditions does not provide substitutions for the ?x variable that is used for representing the policy addressee. This limitation of the model is mainly due to the design choice to propose a model for reasoning about policies that do not create deontic relations when policies are activated.

### Formalizing Norms Inducing Normative Power

The **sixth step** of the methodology consists in understanding whether the norm implies normative power or not, this by taking into account what is explained in “Norms that Induce Normative Powers” section.

Norms represent a normative power when, thanks to the norm, one agent can trigger the creation of an obligation for another agent. For example, in the following norm (that we call Norm3) “when professors ask their PhD students to write a paper for a conference, the students are obliged to do it before the deadline of the conference”, the agents playing the role of professors have a specific normative power over their PhD students. Conversely, if the activation of the norm creates an obligation for the same agent whose action led to the activation of the norm, the norm does not give any normative power to agents with a given role. For example, Norm1, according to which if an agent enters a restricted traffic zone, they become obliged to pay 5 euro to the City of Milan, does not represent any normative power.

After understanding whether or not the norm represents a normative power, the norm designer must figure out how this power is represented and, based on this, select the appropriate model to represent the norm. As described in “Norms that Induce Normative Powers” section, there are two types of such norms. Firstly, there are norms that have the notion of *power* explicitly in the description of the norm, and secondly, there are norms that induce normative power for agents who have a certain role but do not explicitly mention the notion of normative power in their text.

If the notion of power is explicit in the text of the norm, a model of the notion of normative power, such as that proposed in [30], is required for the formalization of the norm. For example, this would happen for the formalization of the following norm: “When Professor John asks a PhD student to write a conference paper and they have the normative power to oblige his PhD students to write a conference paper, the student becomes obliged to write the paper”.

On the other hand, if the norm is stated in such a way that it does not require an explicit formalization of the concept of normative power as in the case of Norm3, then it is possible to model it with a norms model, such as the T-Norm, without using a model of normative power. Indeed, Norm3 can be formalized using the T-Norm model as:
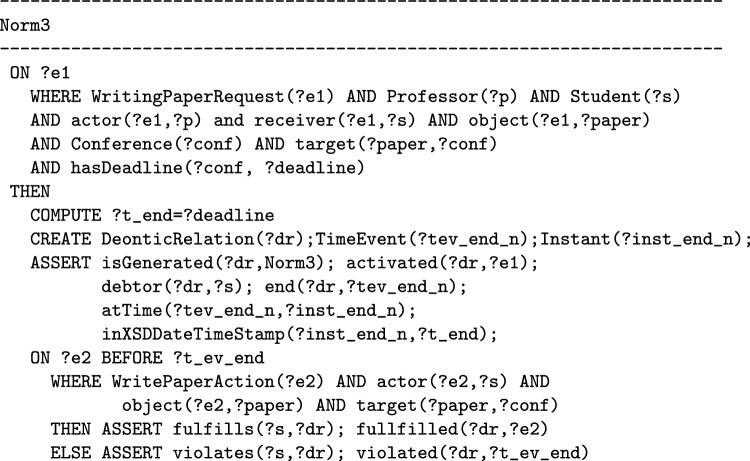


### Representing Permissions and Exemptions

In this section, we describe how to formalize those norms that express the deontic notion of *permission* and *exemption*. The meaning of having a permission to do something has been extensively researched in the literature, and several forms of permission have been analyzed. In [35, 36], they consider permissions as an exception to prohibitions, which means that the agent’s behavior does not count as a violation and thus there will be no penalty if the permitted action happens. In many normative systems there is a default assumption related to permissions and prohibitions, i.e. when it is necessary to explicitly specify permission to perform an action, it is because a prohibition to perform that action already exists or it is assumed to exist. Indeed, it is possible to distinguish among two types of permissions: *weak permission* and *strong permission*. The important distinction between strong and weak permission has been addressed in [37]. If there is no prohibition to do an action, it means that we have weak permission to do that action. On the other hand, if there exists explicit permission for doing a prohibited action, we call it strong permission. In a similar situation for obligations, if an agent has strong permission to derogate an obligation, it is called an *exemption*.

The notion of permission is formalized in different ways in the various models of norms considered in this work. In the T-Norm model the notion of *exception* can be used for formalizing both strong permissions (exceptions to prohibitions) and exemptions (exceptions to obligations) as will be presented below. Differently, in OWL-POLAR weak permissions are simply represented with a *P* indicating that the policy is a permission, all other policy parameters are those already discussed for the specification of obligations or prohibitions. The model does not provide a way to indicate that a permission allows an action that is prohibited. Similarly, in ODRL, permissions are formalised within a policy using the permission property and “a Permission allows an action, with all refinements satisfied, to be exercised on an Asset if all constraints are satisfied and if all duties are fulfilled”.^17^ For instance, it is possible to specify that an agent is allowed to play a game for a certain time interval and it is impossible to indicate what is the precise prohibition to which an exception is being created.

When a prohibition or obligation is formalised using the T-Norm model, it is possible to introduce exceptions to it using one of the following types, whose abstract model was introduced in “The T-Norm Model” section:

1. Exception to norms activation;
2. Exception to deontic relations (that were generated by the activation of a norm) due to some particular conditions on the event that is regulated by the norm;
3. Exception to specific deontic relations due to an event that is not the one regulated by the norm.

Exceptions of the **first type** are used to block the activation of a norm in certain specific cases concerning the conditions of activation of the norm. For example, one may wish to inhibit the activation of Norm1 if the vehicle entering the limited traffic area is an ambulance. In the specification of the exception, it is enough to write the further conditions that ?e1 must satisfy, knowing that when the exception is evaluated, the other conditions on ?e1 contained in Norm1 will also be considered. The exception is able to inhibit the activation of the norm because the constructs specifying exceptions are all evaluated before the constructs used to represent the norms in a system. The exception to Norm1 described above is formalised as follows:
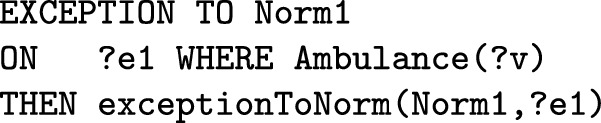


Where the specific exception to Norm1 for the specific event is created if the vehicle that satisfies the activation condition of Norm1 (represented with the variable ?v), by entering in the limited traffic area, belongs to the class Ambulance.

The **second type** of exception is used to inhibit the generation of the violation or fulfilment of deontic relations even if the action regulated by the norm is performed or not performed. For example, an exception to the prohibition expressed by Norm2 may be to give teachers permission to borrow DVDs from the library for educational purposes even if the two years have not elapsed. In the specification of the exception, it enough to add that the recipient of the lending action introduced in the formalisation of Norm2 (see “Representing the Temporal Constraints of Norms” section) by using the variable ?e2, belongs to the class Teacher. This exception is expressed with the T-Norm model as follows:
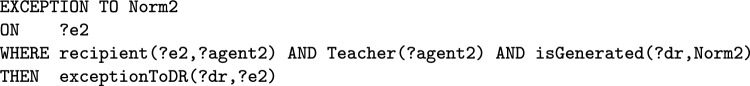


The **third type** of exception is used to inhibits the fulfillment and violation of *specific deontic relations*. Due to the fact that these exceptions are brought about by events that are not the one regulated by the norm, they differ from those of the second kind. For instance, an exception to the following Covid-19 norm: “a person who has a positive swab to Covid-19 cannot leave the house for the next 15 days”, is: “if the house is on fire then everybody is allowed to leave it”. This exception is activated by an event (the house is on fire) that is different from the action that is regulated by the norm (leaving the house). We model this exception in the following way:
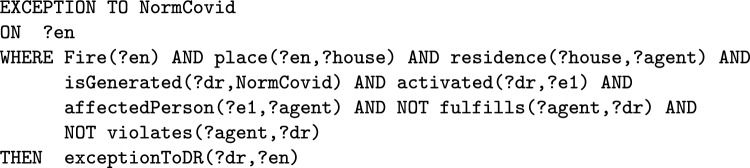


## Conclusions

Nowadays, the importance of formalizing the rules, norms and policies that govern activities of human and software agents in order to make them understandable and processable by machines, encourages many research communities to propose formal models or languages to reach this goal. In this paper, we proposed a methodology that explains how a norm designer can formalize norms written in a natural language into a machine-readable format by understanding the type of the norm under consideration, choosing the appropriate model, and using it correctly.

The presentation of this paper has two objectives. Firstly, proposing and discussing a methodology that can be used by people to translate norms written in natural language into a language specifically designed for the formal specification of norms. The second goal is to propose and discuss a rich set of norm types that could be used in the steps of the proposed methodology, to study the expressive power of different formal models of norms, to compare them, and to design methods for translating norms written in one formal language into norms written in another formal language enhancing the interoperability of normative systems.

The proposed methodology is relevant mainly for two reasons. The first is that it introduces some criteria for the choice of the model of norms to be adopted for the formalization of a given norm. For example, one of the criteria discussed is whether the norm is activated by the occurrence of an event or by the occurrence of a state of affairs; another criterion is the presence in the norm of a deadline within which a certain obliged action must be performed. The second is that it is not at all obvious how a norms model can be used to formalize existing norms written in natural language and how to choose between different possible formalizations of the same norm, e.g. deciding whether it is better to formalize a norm as a prohibition to do an action or as an obligation to do the opposite action. The value of the proposed methodology has been highlighted by discussing the formalization with the T-Norm model of two different types of running examples, by presenting the formalization of a norm that induces a normative power and by exemplifying how permissions can be formalized as exceptions to prohibitions.

An interesting characteristic of the proposed methodology is that it is difficult to automate because it requires a deep understanding of the meaning of a norm and requires the user to be able to choose between different ways of formulating the same norm e.g. as an obligation or as a prohibition as discussed in “Representing Obligations and Prohibitions” section.

A limitation of the proposed methodology is that the evaluation of its comprehensibility and feasibility of application for the formalisation of different types of norms has been carried out by a restricted group of researchers. The difficulty in this type of evaluation lies in the fact that in order to be able to use a formal model of norms based on semantic web technologies, it is necessary to have good knowledge in different fields, from the legal one (to understand the meaning of the norm) to the computer field and in particular in the field of normative systems and semantic web technologies (to be able to use formal ontologies), which is not easy to find in a significantly large group of people.

Another limitation of the proposed methodology is that it focuses only on manually transforming norms in natural language to machine-readable norms. We have not investigated how this process can be, at least in part, automated by, for example, trying to extract the meaning of certain parts of the text (e.g. the activation condition of a norm) with machine learning techniques, especially those based on Natural Language Processing techniques as it is discussed by the “AI and Law” community [38].

In our future work, we plan to extend the methodology by discussing the formalization of norms and investigating the possibility of adding more types to our type selection. For example we plan to investigate the formalization of sanctions for norms violations and the formalization of constitutive norms. In addition, we plan to test our methodology with a significant number of developers (students or colleagues who are familiar with this field of research) to check the feasibility of our approach and after receiving their feedback, apply the required modification for the improvement of the methodology. In our future work we plan also to study the applicability of the proposed methodology to other models of norms and in particular to those proposed in the legal field where the “AI and Law” community has proposed interesting legal ontologies and legal rule languages. We also plan to investigate how to extend the T-Norm model so that it can be used for formalizing norms activated by states of affairs.

## Footnotes

1 https://www.w3.org/TR/odrl-model/.

2 https://www.w3.org/TR/odrl-vocab/.

3 In some models the term policy is used, in others the term norm is used. For this reason, the two terms will be used as synonyms in this document.

4 https://www.w3.org/TR/odrl-model/.

5 https://www.oasis-open.org/committees/legalruleml/.

6 https://www.comune.milano.it/aree-tematiche/mobilita/area-c.

7 According to Art. 69 c.1 of the Copyright Law (22-4-1941, no. 633).

8 The ON clause has been chosen instead of the more common IF clause to highlight that the part after THEN is executed when a particular event or action occurs and not simply when a condition is satisfied.

9 The T-Norm ontology in OWL is available at https://raw.githubusercontent.com/fornaran/T-Norm-Model/main/tnorm.owl.

10 The choice of using conjunctions or sequences of atomic assertions (analogously to what is proposed in OWL-POLAR to express the various components of their norm model) has the advantage of avoiding requiring the user of the model to learn a specific formal language, once written those expressions can be easily and automatically translated into the conditions or actions of production rules or into SPARQL queries.

11 https://www.w3.org/TR/odrl-vocab/#vocab-common.

12 Where ?event1 is shortened to ?e1.

13 https://www.w3.org/TR/owl-time/.

14 https://schema.org/.

15 The term “norm designer” refers to the person in charge of formalizing norms with a formal model.

16 The formalization of Norm1 using the T-Norm model is available in [6].

17 https://www.w3.org/TR/odrl-model/#permission.

## References

[1] Fornara N, Colombetti M. Specifying and enforcing norms in artificial institutions. In: Baldoni M, Son TC, Riemsdijk MB, Winikoff M, editors. Declarative agent languages and technologies VI. Berlin, Heidelberg: Springer; 2009. p. 1–17.

[2] Oltramari A, Piraviperumal D, Schaub F, Wilson S, Cherivirala S, Norton TB, Russell NC, Story P, Reidenberg JR, Sadeh NM. Privonto: a semantic framework for the analysis of privacy policies. Semant Web. 2018;9(2):185–203. 10.3233/SW-170283.

[3] Sensoy M, Norman TJ, Vasconcelos WW, Sycara KP. OWL-POLAR: a framework for semantic policy representation and reasoning. J Web Semant. 2012;12:148–60. 10.1016/j.websem.2011.11.005.

[4] Padget J, Vos MD, Page CA. Deontic sensors. In: Proceedings of the Twenty-Seventh International Joint Conference on Artificial Intelligence, IJCAI-18. Vienna: International Joint Conferences on Artificial Intelligence Organization; 2018. p. 475–81. 10.24963/ijcai.2018/66.

[5] Fornara N, Colombetti M. Using semantic web technologies and production rules for reasoning on obligations, permissions, and prohibitions. AI Commun. 2019;32(4):319–34. 10.3233/AIC-190617.

[6] Fornara N, Roshankish S, Colombetti M. A framework for automatic monitoring of norms that regulate time constrained actions. In: Proceedings of the international workshop on Coordination, Organizations, Institutions, Norms and Ethics for Governance of Multi-Agent Systems (COINE), co-located with AAMAS 2021, 3rd May 2021, London, UK, 2021. arXiv, New York; 2021. 10.48550/ARXIV.2105.00200. arXiv:2105.00200.

[7] Fornara N, Sterpetti M. An architecture for monitoring norms that combines OWL reasoning and forward chaining over rules. In: al., E.M.S., editor. Proceedings of the Joint Ontology Workshops 2021 Episode VII: the Bolzano Summer of Knowledge co-located with the 12th International Conference on Formal Ontology in Information Systems (FOIS 2021), and the 12th International Conference on Biomedical Ontologies (ICBO 2021), Bolzano, Italy, September 11–18, 2021. CEUR Workshop Proceedings, vol. 2969. CEUR-WS.org, Aachen; 2021. http://ceur-ws.org/Vol-2969/paper8-DEMO.pdf.

[8] Roshankish S, Fornara N. A methodology for formalizing different types of norms. In: Baumeister D, Rothe J, editors. Multi-Agent Systems—19th European Conference, EUMAS 2022, Düsseldorf, Germany, September 14–16, 2022, Proceedings. Lecture Notes in Computer Science, vol. 13442. Cham: Springer; 2022. p. 348–363. 10.1007/978-3-031-20614-6_20.

[9] Andrighetto G, Governatori G, Noriega P, van der Torre LWN, editors. Normative multi-agent systems. Dagstuhl Follow-Ups, vol. 4. Schloss Dagstuhl - Leibniz-Zentrum für Informatik, Germany; 2013. http://drops.dagstuhl.de/opus/portals/dfu/index.php?semnr=13003.

[10] Chopra A, van der Torre L, Verhagen H, Villata S, editors. Handbook of normative multiagent systems. UK: College Publications; 2018.

[11] Steyskal S, Polleres A. Towards formal semantics for ODRL policies. In: Bassiliades N, Gottlob G, Sadri F, Paschke A, Roman D, editors. RuleML 2015, Berlin, Germany, August 2–5, 2015, Proceedings. Lecture Notes in Computer Science, vol. 9202. Cham: Springer. 2015; p. 360–75. 10.1007/978-3-319-21542-6_23.

[12] Gandon F, Governatori G, Villata S. Normative requirements as linked data. In: Wyner AZ, Casini G, editors. Legal Knowledge and Information Systems—JURIX 2017: the Thirtieth Annual Conference, Luxembourg, 13–15 December 2017. Frontiers in Artificial Intelligence and Applications, vol. 302. Amsterdam: IOS Press; 2017. p. 1–10. 10.3233/978-1-61499-838-9-1.

[13] Benzmüller C, Parent X, van der Torre L. Designing normative theories for ethical and legal reasoning: Logikey framework, methodology, and tool support. Artif Intell. 2020;287: 103348. 10.1016/j.artint.2020.103348.

[14] Uszok A, Bradshaw J, Jeffers R, Suri N, Hayes P, Breedy M, Bunch L, Johnson M, Kulkarni S, Lott J. Kaos policy and domain services: toward a description-logic approach to policy representation, deconfliction, and enforcement. In: Proceedings POLICY 2003. IEEE 4th international workshop on policies for distributed systems and networks; 2003. p. 93–6. 10.1109/POLICY.2003.1206963.

[15] Kagal L. A policy-based approach to governing autonomous behavior in distributed environments. Ph.D. thesis, University of Maryland Baltimore County, Baltimore MD 21250. Department of Computer Science and Electrical Engineering. 2004.

[16] Bonatti P, Olmedilla D. Driving and monitoring provisional trust negotiation with metapolicies. In: Sixth IEEE International Workshop on Policies for Distributed Systems and Networks (POLICY’05). 2005. p. 14–23. 10.1109/POLICY.2005.13.

[17] Bonatti PA, Olmedilla D. In: Antoniou G, Aßmann U, Baroglio C, Decker S, Henze N, Patranjan P-L, Tolksdorf R, editors. Rule-based policy representation and reasoning for the semantic web. Berlin, Heidelberg: Springer; 2007. p. 240–68. 10.1007/978-3-540-74615-7_4.

[18] De Vos M, Kirrane S, Padget J, Satoh K. ODRL policy modelling and compliance checking. In: Fodor P, Montali M, Calvanese D, Roman D, editors. Rules and reasoning. Cham: Springer; 2019. p. 36–51.

[19] Padget J, ElDeen Elakehal E, Li T, De Vos M. In: Aldewereld H, Boissier O, Dignum V, Noriega P, Padget J, editors. InstAL: an Institutional Action Language. Cham: Springer; 2016. p. 101–24. 10.1007/978-3-319-33570-4_6.

[20] Governatori G, Iannella R. A modelling and reasoning framework for social networks policies. Enterp Inf Syst. 2011;5(1):145–67. 10.1080/17517575.2010.513014.

[21] Garcia-Camino A, Noriega P, Rodriguez-Aguilar JA. Implementing norms in electronic institutions. In: Proceedings of the Fourth International Joint Conference on Autonomous Agents and Multiagent Systems. AAMAS ’05. New York: ACM; 2005. p. 667–73. 10.1145/1082473.1082575.

[22] Álvarez-Napagao S, Aldewereld H, Vázquez-Salceda J, Dignum F. Normative monitoring: semantics and implementation. In: Vos MD, et al, editors. COIN@AAMAS 2010, Toronto, Canada, May 2010, COIN@MALLOW 2010, Lyon, France, August 2010, Revised Selected Papers. LNCS, vol. 6541. Berlin: Springer. 2010. p. 321–36. 10.1007/978-3-642-21268-0_18.

[23] Athan T, Boley H, Governatori G, Palmirani M, Paschke A, Wyner A. Oasis legalruleml. In: Proceedings of the Fourteenth International Conference on Artificial Intelligence and Law. ICAIL ’13. New York: Association for Computing Machinery; 2013. p. 3–12. 10.1145/2514601.2514603.

[24] Serramia M, Lopez-Sanchez M, Rodriguez-Aguilar JA, Morales J, Wooldridge M, Ansotegui C. Exploiting moral values to choose the right norms. In: Proceedings of the 2018 AAAI/ACM Conference on AI, Ethics, and Society. AIES ’18. New York: Association for Computing Machinery; 2018. p. 264–70. 10.1145/3278721.3278735.

[25] Serramia M, López-Sánchez M, Rodríguez JA. A qualitative approach to composing value-aligned norm systems autores. 2020. 10.5555/3398761.3398904. Funded by AI4EU(H2020-825619), H2020-769142, PGC2018- 096212-B-C33, 19S01329- 001 by Ajuntament de Barcelona (Fundació Solidaritat UB).

[26] Morales J, Lopez-Sanchez M, Rodriguez-Aguilar JA, Wooldridge M, Vasconcelos W. Automated synthesis of normative systems. In: Proceedings of the 2013 International Conference on Autonomous Agents and Multi-Agent Systems. AAMAS ’13. Richland: International Foundation for Autonomous Agents and Multiagent Systems; 2013. p. 483–90.

[27] Morales J, Wooldridge M, Rodríguez-Aguilar JA, López-Sánchez M. Off-line synthesis of evolutionarily stable normative systems. Auton Agent Multi-Agent Syst. 2018;32(5):635–71. 10.1007/s10458-018-9390-3. 30147433 10.1007/s10458-018-9390-3 PMC6097804

[28] Dignum F, Broersen J, Dignum V, Meyer J-J. Meeting the deadline: why, when and how. In: Hinchey MG, Rash JL, Truszkowski WF, Rouff CA, editors. Formal approaches to agent-based systems. Berlin, Heidelberg: Springer; 2005. p. 30–40.

[29] Jones AJI, Sergot M. A formal characterisation of institutionalised power. Logic J IGPL. 1996;4(3):427–43. 10.1093/jigpal/4.3.427.

[30] Oren N, Luck M, Miles S. A model of normative power. In: Hoek W, Kaminka GA, Lespérance Y, Luck M, Sen S, editors. 9th International Conference on Autonomous Agents and Multiagent Systems (AAMAS 2010), Toronto, Canada, May 10–14, 2010, vol. 1–3. Richland: IFAAMAS; 2010. p. 815–22. https://dl.acm.org/citation.cfm?id=1838315.

[31] Savarimuthu BTR, Cranefield S, Purvis M, Purvis MK. Identifying conditional norms in multi-agent societies. In: Vos MD, Fornara N, Pitt JV, Vouros GA, editos. Coordination, Organizations, Institutions, and Norms in Agent Systems VI—COIN 2010 International Workshops, COIN@AAMAS 2010, Toronto, Canada, May 2010, COIN@MALLOW 2010, Lyon, France, August 2010, Revised Selected Papers. Lecture Notes in Computer Science, vol. 6541. Berlin, Heidelberg: Springer; 2010. p. 285–302. 10.1007/978-3-642-21268-0_16.

[32] Demolombe R, Louis V. Norms, institutional power and roles: towards a logical framework. In: Esposito F, Ras ZW, Malerba D, Semeraro G, editors. Foundations of Intelligent Systems, 16th International Symposium, ISMIS 2006, Bari, Italy, September 27–29, 2006, Proceedings. Lecture Notes in Computer Science, vol. 4203. Berlin, Heidelberg: Springer; 2006. p. 514–23. 10.1007/11875604_58.

[33] Wright GH. Deontic logic. Mind New Ser. 1951;60(237):1–15.

[34] Panagiotidi S, Alvarez-Napagao S, Vázquez-Salceda J. Towards the norm-aware agent: bridging the gap between deontic specifications and practical mechanisms for norm monitoring and norm-aware planning. In: Revised Selected Papers of the COIN 2013, vol. 8386. Cham: Springer; 2014. p. 346–63. 10.1007/978-3-319-07314-9_19.

[35] Boella G, Torre L. Permission and authorization in normative multiagent systems. In: Proceedings of the 10th International Conference on Artificial Intelligence and Law. ICAIL ’05. New York: Association for Computing Machinery; 2005. p. 236–7. 10.1145/1165485.1165526.

[36] Boella G, Torre L. Regulative and constitutive norms in normative multiagent systems. In: Proceedings of the Ninth International Conference on Principles of Knowledge Representation and Reasoning. KR’04. California: AAAI Press; 2004. p. 255–65.

[37] Governatori G, Olivieri F, Rotolo A, Scannapieco S. Computing strong and weak permissions in defeasible logic. J Philos Log. 2013;42(6):799–829. 10.1007/s10992-013-9295-1.

[38] Villata S, Araszkiewicz M, Ashley KD, Bench-Capon TJM, Branting LK, Conrad JG, Wyner A. Thirty years of artificial intelligence and law: the third decade. Artif Intell Law. 2022;30(4):561–91. 10.1007/S10506-022-09327-6.

